# Cancer Courts Immune Response to Aid Growth

**DOI:** 10.1371/journal.pbio.1001004

**Published:** 2010-12-14

**Authors:** Robin Mejia

**Affiliations:** Freelance Science Writer, Berkeley, California, United States of America

**Figure:**
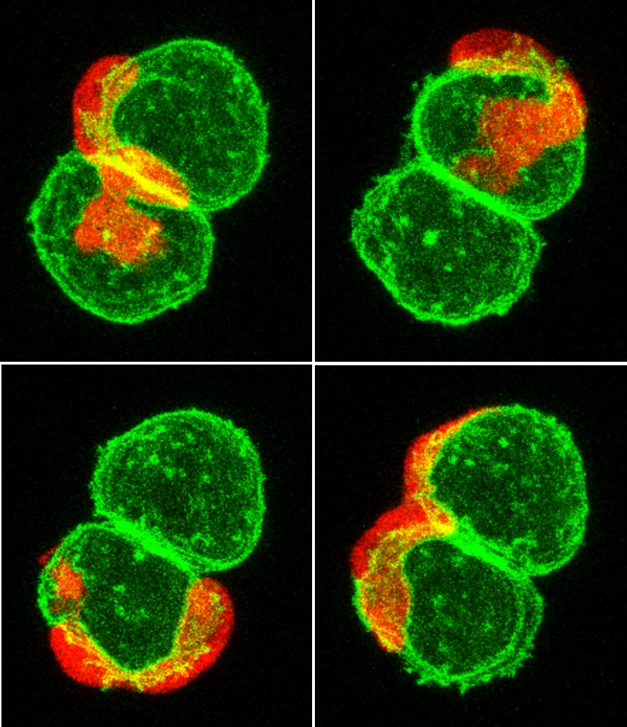
Destroyer or facilitator? An immune cell (red) glides over a doublet of V12Ras-transformed mucus-secreting cells, possible precursors of tumors, in a translucent, 3-day-old zebrafish larva.

In recent years, research has delved into the ways the body's immune system sometimes promotes disease rather than stifling it. Take inflammation. When you cut yourself, the red puffiness that ensues, called acute inflammation, is the body's way of signaling that something's gone wrong and help is needed. If all goes well, various immune cells move in, destroying any pathogens that might enter the wound and helping to repair the damaged tissue. As you heal, the inflammation subsides. However, much like stress—a natural response to crisis that is unhealthy as a steady state—inflammation appears to be useful in the short-term but bad over the long haul. Chronic low-level inflammation has been fingered as a root cause of many diseases, contributing to conditions from diabetes to cancer.

Research in mice has demonstrated that immune cells interact extensively with tumors, and not just to fight them off. Rather, cancers sometimes co-opt the immune system. For example, macrophages help guide metastasis, the spread of cancer cells from advanced tumors to other sites in the body. However, in mice it's been difficult to assess how the immune system interacts with the earliest stages of tumor development.

When an oncogene (a cancer-promoting gene) is activated or a tumor suppressor function lost, a cell can start to grow and divide faster than its neighbors. Eventually, transformed cells overtake the surrounding tissue and form tumors. A new animal study by Yi Feng and colleagues in the UK and Italy illuminates how single, newly-transformed cancer cells engage the body's immune response. Rather than mice, the team used zebrafish, which conserve many of the molecular and cellular components of tumor formation seen in mammals. Moreover, zebrafish larvae offer the advantage of being translucent, allowing investigators to live-image the very beginnings of tumors, when only one or two cells have been transformed.

Using zebrafish that had fluorescently labeled leukocytes, the team used several different tricks to express the human oncogene HRAS in early stage embryos. The oncogene was labeled with a different colored fluorescent tag and engineered to be switched on in melanocytes, specific skin pigment cells, only. The researchers then monitored the first hours and days of development. As the embryo grew, some of the cells were transformed by HRAS, and those transformed cells actively attracted the innate immune cells. The researchers got the same results when using a different oncogene, SRC, for their experiments and after inserting HRAS into a different cell population, mucus-secreting cells, and continued to see the same immune response.

To investigate the analogy that a tumor resembles a wound, the researchers made a laser cut in the same region of the zebrafish larvae where tumors had been observed and imaged the immune response. Early immune cells responded to the cut in a very similar manner. Both wounds and tumor cells produced H_2_O_2_, and the researchers found that immune cells traveled up the H_2_O_2_ gradient towards the cut or cancer.

However, in the case of the tumor, the inflammatory response never resolved, and researchers were able to visualize competing immune responses. Neutrophils and macrophages appeared to engulf cancerous cells, in line with the traditional “search and destroy” conception of immune response. However, other cells formed cytoplasmic tethers linking them to cancerous cells, and in some cases the cancerous cells appeared to drag leukocytes back when they started to leave the region. The tumors resembled chronic skin lesions more than acute cuts, supporting the common analogy that “a tumor is a wound that doesn't heal.”

Still, the researchers wanted to know whether the tumor was avoiding destruction or actually co-opting the immune cells in these earliest stages of development. To test this, they blocked the immune response in three different ways: they prevented the development of immune cells for the first three days after fertilization, and, separately, they used two different strategies to limit H_2_O_2_ production. In each case, immune cells failed to migrate to the cancer site. And each time, when the immune response was blocked, fewer cancer cells formed.

By visualizing the earliest interactions between cancer cells and their host environment, the researchers have shown that even from their earliest stages tumors don't just avoid being destroyed by the immune system. Rather, they appear to court an immune response, co-opting the body's innate immune system to aid and abet their growth.

**Feng Y, Santoriello C, Mione M, Hurlstone A, Martin P (2010) Live Imaging of Innate Immune Cell Sensing of Transformed Cells in Zebrafish Larvae: Parallels between Tumor Initiation and Wound Inflammation. doi: 10.1371/journal.pbio.1000562**

